# Macroeconomic antecedents of racial disparities in psychiatric-related emergency department visits

**DOI:** 10.3389/fpsyt.2024.1287791

**Published:** 2024-06-12

**Authors:** Parvati Singh

**Affiliations:** Division of Epidemiology, College of Public Health, The Ohio State University, Columbus, OH, United States

**Keywords:** economic downturns, psychiatric-related ED visits (PREDVs), help-seeking, emergency department, racial disparities

## Abstract

**Objective:**

To test whether monthly declines in aggregate employment precede a rise in African American psychiatric-related ED visits (PREDVs) relative to white visits among low-income, working-age populations.

**Design:**

This study used repeated cross-sectional time series data for 6.7 million PREDVs among African Americans and white individuals from the State Emergency Department Database in 48 Metropolitan Statistical Areas (MSAs) across four states (Arizona, California, New York, New Jersey) from 2006 to 2011. MSA-level monthly employment data were obtained from the US Bureau of Labor Statistics. The outcome was specified as the race of a PREDV (African American = 1, white = 0). The exposure was operationalized as monthly percent change in MSA-level aggregate employment lagged by 0 to 3 months. Analysis included logistic regressions with county, month and year fixed effects, and clustered standard errors to examine the relation between odds of an African American PREDV (relative to white) following 0 to 3 months lag of MSA-level aggregate employment change.

**Findings:**

Logistic regression results indicate that the odds of PREDVs for publicly insured, working-age African Americans (relative to white individuals) increase 3 months after ambient employment decline (OR: 0.994, 95% CI: [0.990 0.998]).

**Conclusion:**

Economic downturns may marginally increase psychiatric help-seeking in EDs among publicly insured (low-income), working-age African Americans relative to white individuals. Findings from this study may contribute to the theoretical understanding of dynamic drivers of racial disparities in psychiatric ED visits.

## Introduction

Emergency departments (EDs) are not considered ideal for treating psychiatric illnesses as they provide episodic care for conditions that need continuous case management (1). Most EDs have inadequate systematic linkage and follow-up mechanisms to ensure continuity of care post discharge (2) and thus may not confer long-term benefits to psychiatric patients. High ED reliance for mental health portends inferior health outcomes and greater costs for both patients and hospital systems (3).

In the US, African Americans utilize EDs at a disproportionately higher rate for routine psychiatric care relative to all other races and ethnicities (4) even though population-level epidemiological estimates do not find a greater prevalence of mental illness in this group compared with others (5). This racial disparity in psychiatric-related ED visits (PREDVs) for mental health derives from several static and dynamic factors. Static factors that generally do not change over time include low access, poor quality of care when accessed, sociocultural barriers to help-seeking, and racial discrimination within the healthcare system (4). Dynamic factors, on the other hand, tend to vary over time and place. Some examples of dynamic factors that may correspond with differentially greater PREDVs in African Americans relative to others are exogenous changes such as economic downturns (4).

Economic downturns are particularly salient for African Americans as this group is especially vulnerable to the consequences of a declining economy. African Americans form a vulnerable group that is often the first to be laid off during macroeconomic decline (6, 7). Persistently high poverty, lack of financial buffers (income, assets), and entrenched socio-structural disadvantages (disadvantaged neighborhoods, low upward mobility) place a heavy burden on African Americans coping with a contracting economy (8, 9). During the Great Recession of 2008, one in four African Americans reported job loss and the unemployment rate among African American men exceeded that of all other race/ethnicities in the US (10, 11). Construction and manufacturing sectors were the worst affected, and the collapse of these sectors corresponded with greater loss of employment and wages among African Americans relative to other groups (11, 12). African Americans had a steeper reduction in income and were more likely to lose health insurance compared with white individuals (13–17). During this time period, loss of income and health insurance appears to have reduced access to and utilization of routine mental health services, particularly among African American men (18) that, in turn, may exacerbate preexisting conditions and increase treatment costs (19, 20).

Given their differential exposure and vulnerability to macroeconomic downturns, it is plausible that mental health outcomes may worsen among African Americans, relative to other race/ethnicities. Research predating the 2008 recession finds increased psychiatric emergency visits among low-income (publicly insured) African American children following state-level macroeconomic contraction, plausibly owing to greater financial pressure among low-income families and increase in familial strain during periods of high employment uncertainty (21). Studies also suggest that mentally ill African Americans may face reduced societal tolerance and increased risk of involuntary psychiatric commitment, despite relatively higher cognitive functioning (compared with white individuals), during economic downturns (22). Research on psychiatric help-seeking among African Americans during the recent Great Recession, however, remains scarce.

Economic downturns are considered population-level or ecological stressors as they increase stress from income and job loss among the directly affected, as well as uncertainty, fear, and anxiety of potential job loss among those who remain employed (23). For populations that are not laid off during recessions, studies suggest a rise in working hours, reduction in pay, and increased stress due to future uncertainty of employment (24). Individuals who fear job loss significantly outnumber those who are laid off during economic downturns, and periods of economic decline induce fear and anxiety regardless of employment status (25, 26). During such times, populations may experience adverse mental health outcomes and exhibit greater psychiatric care-seeking, manifested as higher number of mental health-related visits to healthcare providers (27). Macroeconomic downturns may increase help-seeking for mental health through multiple mechanisms such as prophylactic care-seeking (preemptive help-seeking to prevent illness), provocation (development of new illness), uncovering (exacerbation of preexisting psychiatric conditions), and cost-shifting (increased reliance on safety nets for mental health) (27, 28). Prior research finds an increase in help-seeking through greater hospital in-patient admissions and psychiatric-related ED visits (PREDVs) within 3 months following macroeconomic contractions (21, 29, 30). These trends may concentrate among low SES groups that do not have sufficient financial and social assets to weather sudden macroeconomic shocks (8).

The age group of 18 to 64 years overlaps closely with the US civilian labor force and may exhibit particularly acute psychiatric responses to abrupt decline in labor demand during economic recessions (8). In addition to employment-related “direct” stressors, low-income, working-age populations experiencing financial insecurity also face indirect stressors such as increased familial strain (31), reduction in resources for caregiving to children, elderly, and the infirm (32), and loss of psychosocial assets (33, 34). These stressors are compounded for low SES working-age African Americans who find themselves shouldering many competing responsibilities but possess limited financial means to purchase substitute care and augment income loss following economic decline (35). In such times, they may turn to the only safety net in the country that is required, by law, to provide care to every visitor regardless of their ability to pay: the Emergency Department. Thus, owing to their differentially greater exposure and vulnerability to economic downturns, low SES working-age African Americans may exhibit greater help-seeking manifested through increased rates of PREDVs following ambient economic decline.

This paper examines whether help-seeking in EDs for mental health, measured through PREDVs, increases among low SES African Americans relative to white individuals, following economic downturns, in the context of the 2008 recession. To our knowledge, only one prior study assesses racial disparities in PREDVs following ambient economic shocks (21). Here, the authors examine publicly insured African American youth (aged 5 to 21 years), relative to white youth, in California from July 1999 to April 2008, and observe an increase in African American visits 1 month after increase in state-level mass layoffs (21). However, the authors do not examine working-age adults and do not include the complete time period of the 2008 recession (December 2007 to June 2009) (36). Their aggregate-level analysis is also limited to the state of California and does not control for potential individual-level confounders (e.g., gender) (21). The present study builds upon this prior research by examining all age groups using individual-level data on outpatient (treat-and-release) PREDVs from four US states spanning 2006–2011. Consistent with prior work, this study uses public insurance to approximate low SES (21). We test differential help-seeking for emergency mental health services among African Americans relative to white and hypothesize that ambient economic decline (measured as monthly employment change in a Metropolitan Statistical Area) will precede an increase in the likelihood of PREDVs in African Americans relative to white individuals among publicly insured (low SES) populations but not among those with private insurance. If results support this hypothesis, we then test, within the publicly insured, whether different age groups (<18, 18–64, >65 years) exhibit differential responses to monthly employment change. Here, we hypothesize that owing to its strong link to the labor market, the working-age group of 18 to 64 years will drive any association observed in the first test.

## Methods

### Variables and data

We retrieved psychiatric-related ED visit data for African Americans and white individuals from the State Emergency Department Database (SEDD), the most comprehensive census of ED visits (in participating states) in the USA (Healthcare Cost and Utilization Project (37). The Agency for Healthcare Research and Quality (AHRQ) sponsors SEDD and makes it available for purchase under the Healthcare Cost Utilization Project (HCUP). SEDD includes encounter-level data for all emergency department visits that did not result in hospitalization. Cross-validation studies with hospital-level databases such as the American Hospital Association show that SEDD includes almost 99% of hospitals (38). Given our focus on African-American/white differences and other reports on the low quality of Hispanic ethnicity in SEDD, we excluded all other race/ethnicities from the analysis.

We defined visits for psychiatric help-seeking as psychiatric-related ED visits (PREDVs) such that a psychiatric diagnosis appears within the set of diagnoses provided (Dx1 to Dx25). Appendix Table A1 provides all psychiatric ICD 9 diagnosis codes grouped by categories within the Clinical Classification Software (CCS) that were used for this study (39). We obtained SEDD data for states with uniform monthly reporting of ICD-9 psychiatric diagnoses, race/ethnicity, gender, public/private insurance status, age, and county. SEDD reports four insurance groups—private, public, self-pay/charity, and other. Scholars often infer uninsured status in the SEDD from “self-pay,” “charity,” and “underinsured” indicators, but the insurance variable does not provide unambiguous information on SES. Private and public insurance status, however, permits low (vs. high) SES approximation (21). For these reasons, we restricted the analysis to consistent, standardized insurance groups, i.e., the privately insured (commercial carriers, HMOs, PPOs) and the publicly insured (Medicaid, Medicare) (37). These restrictions yielded four populous states for six years, 2006 to 2011, constituting approximately 20% of US population: Arizona, California, New Jersey, and New York.

We retrieved monthly employment data, at the Metropolitan Statistical Area (MSA) level, from the Bureau of Labor Statistics’ Local Area Unemployment Statistics (BLS-LAUS) database (40). These data are derived from the Current Population Survey (CPS) and form the basis of monthly national employment estimates produced by the Bureau of Labor Statistics since 1990 (41). We used Metropolitan Statistical Area (MSA) as the spatial unit of exposure. MSAs are high population density urban areas that form the “nucleus” of economic activity, often spanning multiple cities or counties (42, 43). They wield significant economic influence over their subregions and have a higher population of African Americans relative to non-metropolitan, rural areas (42, 43). Aggregate employment decline in an MSA indicates that some proportion of that population (primarily among the working-age group of 18 to 64 years) has lost income, and this loss may “ripple” through the local economy. The psychiatric sequelae of a contracting economy are not restricted to those who lose employment but also affect those who remain employed (27). Employment decline percent at the MSA level captures this population-level exposure to ambient economic distress for the unemployed, the under-employed, and the employed.

We operationalize the key independent variable as percent change in monthly employment = (x_m_ – x_m-1_)/(x_m-1_), where x_m_ is the number of people employed in a Metropolitan Statistical Area (MSA) in a particular month and x_m-1_ is the number of people employed in the previous month. This specification yields the percentage change in employment in a given month relative to the previous month. A positive value indicates employment gain, and a negative value signifies aggregate employment decline. Our treatment of this exposure removes secular trend from the employment series. Previous research has used this metric for studying economic contractions as population-level stressors (25, 44). The percent employment change metric also overcomes the drawbacks of the unemployment rate in that it (a) represents immediate change and accounts for inflows, outflows, or changes in the civilian labor force (45) and (b) does not present sizeable directional distortion during brief periods of economic expansion (46–48). Public accessibility of MSA-level employment series also encourages replication and independent verification of our analysis.

We merged the aggregate-level, percent employment change variable (by MSA-month) to psychiatric ED visits from SEDD using county-MSA crosswalk files (49). The analytic period includes a total of 48 MSAs (4 states) for 72 months (2006–2011). In keeping with past research on the induction period of increase in psychiatric symptoms, ED visits, and hospitalizations following ambient changes, we test for perturbations in PREDVs from 0 to 3 months after aggregate employment change (21, 29, 30). This short lag period tests for proximate responses to exposure and avoids potential confounding by other factors that may correlate with economic downturns but take longer to manifest (such as home foreclosures) (50).

### Analysis

We test whether African Americans, more than white individuals, show increased odds of a PREDV following employment decline in that MSA month. We define race as a binary outcome variable (African American = 1; white = 0) to examine whether changes in exposure (area-level monthly employment decline) increase the odds of a PREDV by an African American relative to white. We use public insurance as a surrogate for low SES relative to the privately insured (high SES) (21). Commensurate with theory that predicts greater psychiatric help-seeking and ED reliance among low SES populations (21), we estimate separate models by insurance groups (private, public) to test whether a decline in aggregate (MSA-level) percent employment precedes an increase in the odds of African American ED visits, relative to white, only among the publicly insured. We further stratify these two insurance types by age groups (<18 years, 18 to 64 years, and >64 years). We assume varying levels of exposure to employment change by age group based on their connection to the labor force and test whether groups that do not form a large part of the civilian labor force (i.e.,<18 years and >64 years) do not show as strong an association between exposure and outcome as do individuals most likely to be directly affected by economic decline, i.e., those between 18 to 64 years of age.

I estimate the following logistic regression equation:


(1) π(Yi,c,m,t)1−π(Yi,c,m,t) = exp[β0+ ∑n=14βnXr, m−n+1,t+ β'Ki,c,t,m+ β'γr+ β'm+ β't+ β'γr*L +ε]

where $\;\frac{\text{π}\left(\text{Y}i,c,m,t\right)}{1-\text{π}\left(\text{Y}i,c,m,t\right)}$ is the log-odds of a psychiatric diagnosis occurring in African Americans (Y = 1) relative to white individuals (Y = 0) for ED visit *i* in county *c* during month *m* and year *t*. The set of ∑X*_r,t,m-n+1_* (n = 1 to 4) represents the percent employment change variable X in MSA *r*, month *m*, *m-1* (month lagged by 1), *m-2* (lag 2), *m-3* (lag 3), and year *t*, respectively. **K** is the vector of visit-level characteristics: gender, age, age squared, and insurance status (private, public). Indicator variables for each county, **γ*_r_***, control for time-invariant county (and MSA) level factors that correlate with both MSA-level employment change and race differences in PREDVs. Indicators for month **m** absorb seasonality shared by PREDVs and employment cycles. Year fixed effects, **t**, account for annual policy changes (e.g., expansion of mental health parity under the Affordable Care Act) (51) that may affect the outcome. **γ*_r_*** *L is a vector of county-specific linear time trends that control for unobserved variables that trending linearly over the 72-month study period and may influence racial disparities in psychiatric ED use (e.g., secular trends in PREDVs). We cluster standard errors at the MSA level to account for heteroskedasticity.

For tests that reject the null, we estimate the number of African American PREDVs statistically attributable to a unit increase in exposure in the following manner: (i) First, we obtain odds of African American PREDVs relative to white within one standard deviation of employment change. (ii) Next, we apply the discovered coefficient (odds ratio) of exposure from logistic regression analysis to the odds ratio from step (i) to estimate the additional African American PREDVs corresponding to unit decline in employment change.

Given that we test the individual-level odds of African American PREDVs relative to white individuals, following aggregate monthly percent employment decline, it remains plausible that any hypothesis tests that reject the null may arise from a net *decline* in psychiatric-related ED visits among white individuals, that may mislead inference as a “true” increase in odds among African Americans. To address this concern, we aggregate psychiatric-related ED visit rates (per 100,000 population) by race (African American, white) and test, in a linear fixed effects regression framework, the relation between population rates of PREDVs and percent employment change for each race (separately, for hypothesis tests that reject the null). We also examine aggregate-level racial disparities in PREDVs following economic contraction by interacting the exposure (0 to 3 month lags) with binary race indicator (African American = 1, white = 0), to gauge whether and to what extent individual-level results conform with aggregate-level results (for hypothesis tests that reject the null). Similar to Equation 1, these analyses of aggregate-level trends control for region, month, year fixed effects, state-specific linear time trends, and incorporate heteroscedasticity-robust standard errors.

As a robustness check, we test the consistency of direction of association between the dependent and independent variables in Equation 1 through a linear probability model using OLS regression (with binary outcome) for hypothesis tests that reject the null. We also conduct a test of extreme “employment decline shocks” to gauge whether odds of African American PREDVs following sudden and stark regional economic contractions align with findings from a more continuous treatment of the exposure.

The inclusion of the year 2011 raises an important concern regarding changes in psychiatric ED visits due to insurance coverage expansion under the Affordable Care Act (ACA) (51). After the initial provisions of mental health parity under the ACA took effect in 2010, ED visits rose among the newly insured (52, 53) and among new Medicaid recipients not accepted by primary care physicians (54). Newly insured African Americans may have therefore increased ED utilization for mental health after 2010, as suggested by findings from the Oregon health insurance experiment (55). For this reason, we conduct a sensitivity analysis by estimating Equation 1 for all years excluding 2011 to test whether the association between employment decline and psychiatric ED visits matches results from hypothesis testing. We conduct all analyses with Stata SE (version 14.2) (56).

## Results

**Table 1** shows the descriptive statistics of our analytic sample. Between 2006 and 2011, SEDD includes 6.7 million PREDVs of which nearly 80% are white and 20% are African Americans. The number of visits among the publicly insured is approximately 1.4 times higher than those with private insurance. The most common diagnoses, accounting for nearly 40% of all PREDVs, are mood, anxiety, and alcohol abuse-related disorders. Monthly percent employment change has a mean of −0.04%, indicating a general period of employment decline reaching as low as −17.9%. Overall, the sample has 103 more MSA months with employment decline (percent employment change< 0) than employment gain (percent employment change > 0). Nearly 7% of MSA months with negative employment change show extreme decline, i.e., exceed three standard deviations below the mean.

**Table 1:** Description of key attributes of psychiatric-related ED visitors and 48 MSAs (four states: AZ, CA, NJ, NY), 2006–2011, in the study sample.

Individual-level attributes	African American N (%)	White N (%)
Sample size	1,376,698 (20.4)	5,382,753 (79.6)
Females	728,316 (53)	2,996,646 (56)
Males	648,382 (47)	2,386,107(44)
Private insurance	473,494 (34.4)	2,331,507 (43.3)
Public insurance	903,204 (65.6)	3,051,246 (56.7)
Top 3 diagnoses:		
Mood disorders	416,444 (17.4)	1,892,343 (23)
Anxiety disorders	270,356 (11.3)	1,490,265 (18.1)
Alcohol abuse-related disorders	399,778 (16.7)	1,274,905 (15.5)
MSA-level attributes		
Mean percent employment change (std. dev.)	−0.04 (0.94)	
Range of percent employment change	Minimum: −20.6 Maximum: 17.9	
Total number of MSAs	48	
Total number of counties	96	
Number of MSA-months with employment decline (employment change< 0)	1,575	
Number of MSAs-months with employment gain (employment change > 0)	1,472	
Number of MSA-months with acute employment decline (employment change<−3)	111	

**Figure 1:**
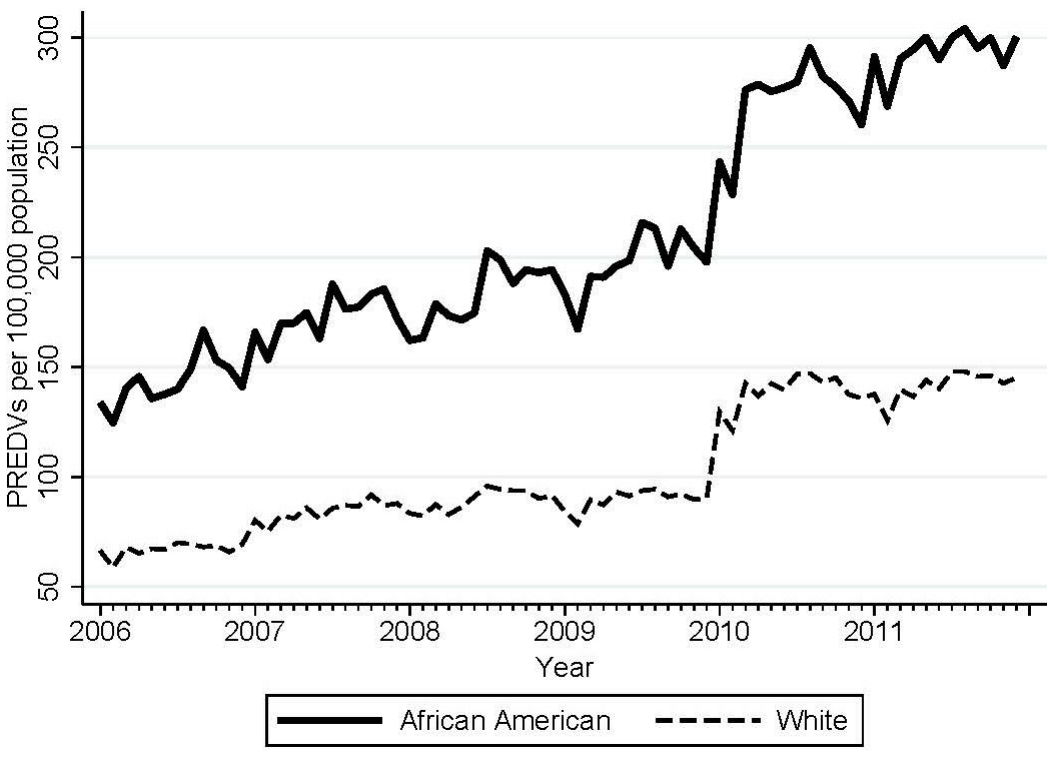
Monthly psychiatric-related ED visits (PREDVs) per 100,000 population for African Americans and white individuals in 48 MSAs (four states: AZ, CA, NJ, NY), 2006–2011, in the study sample.

**Figure 2:**
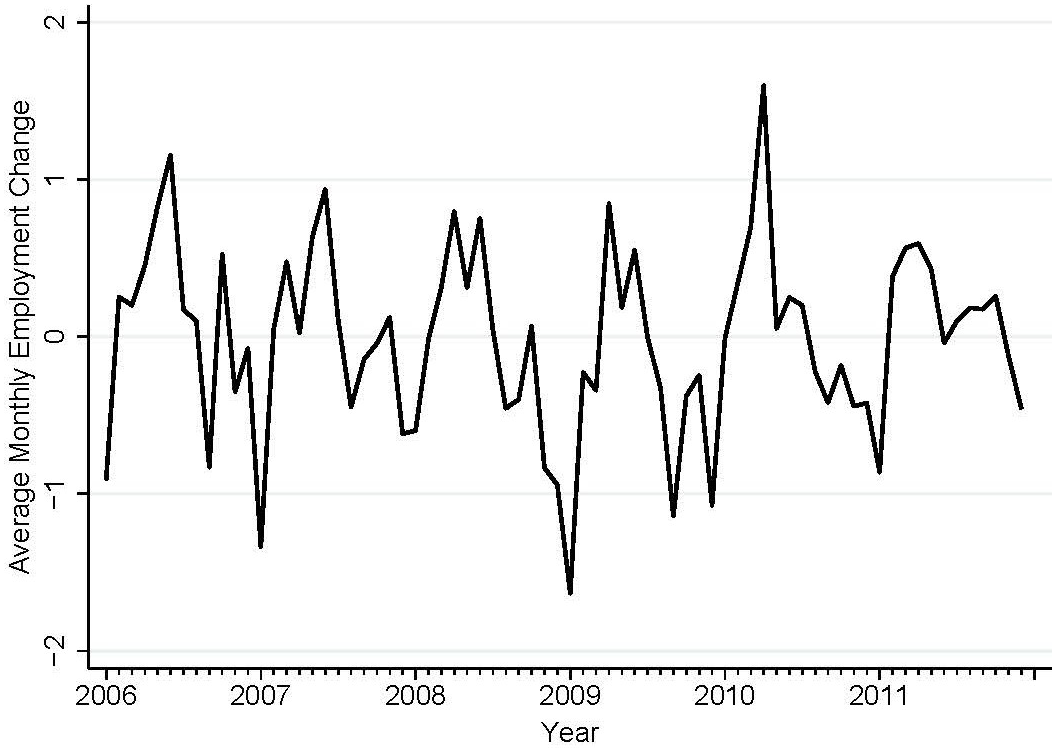
Average monthly percent employment change in 48 MSAs (four states: AZ, CA, NJ, NY), 2006–2011, in the study region.

**Figure 1** shows the trends in PREDVs (per 100,000 population) by race in our study sample. The positive slopes of ED visit rates for both races cohere with national trends in emergency department utilization for psychiatric care over the study period (57, 58). **Figure 2** shows the monthly mean of employment change averaged across 48 MSAs over the study period. In **Figure 2**, positive values (above zero) indicate aggregate employment gain and negative values indicate employment loss.

**Table 2** presents the results of logistic regression analysis for PREDVs among African Americans and white individuals. For the full sample (model a), we fail to reject the null at any of the specified exposure lags. However, among the publicly insured (model b), decline in percent employment change lagged by 3 months precedes an increase in the odds of an African American PREDV relative to white. Here, a unit decrease in employment decline three months prior (m-3) increases the odds of an African American ED visit by 0.5% (compared with white). We observe no relation between percent employment change and the odds of African American PREDVs at any specified lags (0 to 3) for the privately insured population (model c). Female gender is associated with lower odds of African American PREDVs overall and among the privately insured, but not among those with public insurance.

**Table 2:** Logistic regression results predicting African American race (relative to white) among PREDVs as a function of monthly percent employment change lagged by 0 to 3 months (other covariates not shown).

Covariates	*Model a: full sample*		*Model b: public insurance*		*Model c: private insurance*	
	Odds ratio	95% confidence interval	Odds ratio	95% confidence interval	Odds ratio	95% confidence interval
Employment change (lag 0)	0.996	[0.991–1.001]	0.998	[0.993–1.002]	0.991	[0.982–1.001]
Employment change (lag 1)	0.998	[0.994–1.003]	0.999	[0.994–1.003]	0.998	[0.989–1.008]
Employment change (lag 2)	0.998	[0.993–1.002]	0.998	[0.994–1.002]	0.997	[0.987–1.006]
Employment change (lag 3)	0.996	[0.991–1.000]	0.995*	[0.991–0.999]	0.996	[0.987–1.005]
Sex: female (reference = male)	1.073**	[1.026–1.122]	1.034	[0.985–1.085]	1.137***	[1.082–1.195]
Age	1.020***	[1.005–1.035]	1.013	[0.996–1.030]	1.003	[0.984–1.023]
Age squared	1.000***	[0.999–1.000]	0.999***	[0.999–0.999]	0.999	[0.999–1.000]
Public insurance (reference = private)	2.277***	[2.093–2.478]	–	–	–	–
Sample size (N)	6,681,934		3,897,218		2,784,716	

Model a shows results from analysis of unrestricted full sample; Model b shows results among PREDVs with public insurance; Model c shows results among PREDVs with private insurance.

*p value< 0.05; **p value< 0.01; ***p value< 0.001.

**Table 3** presents the results of logistic regression by age groups among the publicly insured. Percent employment change at all lags shows no relation with odds of African American PREDVs (relative to white) for children (model a) and older adults (model c). However, among the 18- to 64-year age group, odds of an African American PREDV increase by 0.6% following decline in employment change lagged at 3 months. The magnitude of association between odds of an African American PREDV and the third lag of monthly employment change is consistent with **Table 2** (Model b) but of slightly greater magnitude.

**Table 3:** Logistic regression results predicting African American race (relative to white) among publicly insured PREDVs as a function of monthly percent employment change lagged by 0 to 3 months (other covariates not shown).

Covariates	*Model a: age<18 years*		*Model b: age 18 to 64 years*		*Model c: age >64 years*	
	Odds ratio	95% confidence interval	Odds ratio	95% confidence interval	Odds ratio	95% confidence interval
Employment change (lag 0)	0.997	[0.986–1.008]	0.997	[0.991–1.0032]	1.002	[0.991–1.012]
Employment change (lag 1)	1.002	[0.986–1.020]	1.001	[0.995–1.006]	0.987	[0.974–1.001]
Employment change (lag 2)	1.008	[0.994–1.022]	0.997	[0.992–1.002]	0.997	[0.985–1.010]
Employment change (lag 3)	0.998	[0.986–1.009]	0.994**	[0.990–0.998]	1.000	[0.987–1.011]
Sex: female (reference = male)	0.986	[0.938–1.037]	1.067*	[1.013–1.124]	1.000	[0.949–1.054]
Age	1.022	[0.969–1.077]	1.004	[0.991–1.016]	0.945	[0.820–1.089]
Age squared	0.998	[0.996–1.001]	1.000*	[0.999–0.999]	1.000	[0.999–1.001]
Sample size (N)	165,945		2,659,531		1,071,742	

Model a shows results from analysis of publicly insured PREDVs aged< 18 years; Model b shows results among publicly insured PREDVs aged 18 to 64 years; Model c shows results among publicly insured PREDVs aged >64 years.

*p value< 0.05; **p value< 0.01; ***p value< 0.001.

Using the coefficient of exposure at lag 3 from **Table 3** (model b), we estimated the number of African American PREDVs statistically attributable to declines in employment change. In our data, a median county-month (based on the ratio of African American to white PREDVs) has 23 African American and 109 white PREDVs (publicly insured, working-age), yielding a base odds ratio of 0.045. Application of the discovered coefficient of exposure at lag 3 (from **Table 3**, model b) to this base rate yields 1.5 additional African American visits per unit decline in employment change. Put another way, we observe 15 additional PREDVs in a median county among low-SES, working-age African Americans, relative to white, 3 months after a 10% decline in MSA-level monthly employment.

Appendix Tables A2–5 present results from robustness and sensitivity checks. Among the 18–64-year-old, publicly insured group, aggregate analyses show a marginal increase in rates (per 100,000 population, log transformed outcome) of psychiatric ED visits 1 month following decline in exposure among white individuals and 3 months after percent monthly employment decline among African Americans (Table A.2, models a, b). Interaction of exposure with binary race shows an increase in population rates of African American psychiatric ED visits, relative to white individuals, at exposure lag 3, which aligns with findings from **Table 3**, model b (Table A.2, model c). Whereas these exposure coefficients do not reach conventional levels of statistical significance, they provide evidence that our original inference does not likely arise from decline in psychiatric ED visits among white individuals alone Table A.2).

Coefficients from OLS regression-based linear probability models (Appendix Table A3) show consistency with results from **Table 3** (model b) in the direction of association between percent employment change and outcome among 18–64-year-olds who are publicly insured. Appendix Table A4 shows logistic regression results of sensitivity analysis after excluding the year 2011. The direction and magnitude of association between percent employment change at lag 3 and odds of African American ED visits (relative to white individuals) is identical to that in **Table 3** (model b) indicating that expansion of mental health parity under initial provisions of the ACA does not account for results from hypothesis tests.

As another sensitivity analysis, we conducted an extremes test of employment decline “shocks”. We converted the exposure to zero for all employment change values greater than −3 (representing three standard deviations below the mean) leaving values less than −3 continuous. We used this exposure specified at lags 0 to 3 to replicate the logistic regression analysis from **Table 3**, model b. Results from this extremes test (Appendix Table A5) cohere with original tests. The association between employment shock at lag 3 and odds of African American PREDV relative to white is over twice that of the exposure at lag 3 in **Table 3**, model b. Per this extremes test, incremental employment decline at the extremes corresponds with a 1.3% increase in an African American PREDV relative to white.

## Discussion

We focused on racial disparities between African Americans and white individuals with respect to PREDVs and tested whether a decline in aggregate employment precedes a rise in the odds of African American visits relative to white individuals over a time period that included the 2008 economic recession. We used high-quality Emergency Department data for 48 Metropolitan Areas (four states) spanning 72 months from 2006 to 2011. Results show that employment decline during the study period corresponds with a modest increase in the odds of publicly insured African American visits relative to white individuals 3 months after exposure. Furthermore, as hypothesized (owing to their close overlap with the civilian labor force), we observe this result only among the working-age population (18–64 years) of publicly insured African Americans. These results remain robust to multiple sensitivity checks.

Strengths of this study include the use of repeated cross-sectional time series data comprising 6.7 million psychiatric ED encounters across a broad range of MSAs over a 5-year period (2006–2011). This time period includes both the Great Recession and years of economic stability. We utilize objectively defined clinical psychiatric ICD 9 diagnoses that reduce measurement errors associated with self-reported data. Brief exposure time lags minimize confounding that may arise from long-run sequelae of economic downturns. We also control for time-invariant regional differences in ED use by including county “fixed effects”. In addition, we control for any patterns over time in ED use across all MSAs by including year and month indicator variables. We know of no study that has longitudinally examined racial disparities in psychiatric help-seeking over the period of the 2008 recession, focusing on low-income, working-age African Americans.

Four potential mechanisms may contribute to psychiatric help-seeking during economic contractions: (i) provocation, (ii) uncovering, (iii) prophylaxis, and (iii) cost shifting (27, 28). The provocation pathway proposes an increase in new disorders following macroeconomic decline due to increased socioeconomic disadvantage and elevation of stressful life events (29). Uncovering posits increased hospitalization among the chronically ill as economic adversity might make it difficult for patients to manage their preexisting conditions due to reduced resources (loss of income, health insurance, inability to continue medications) and increased reporting (arising from reduced tolerance to mental illness or disordered behavior) (59). Prophylaxis encompasses increase in utilization of mental healthcare facilities either in anticipation of, or as a risk-averse response to financial uncertainty (60). Cost shifting predicts that following income or job loss, people may “shift” from private treatment to public facilities and safety nets, such as EDs, for psychiatric care (61). Collectively, these help-seeking pathways may underlie differentially greater PREDVs among African Americans compared with other groups during economic downturns (60, 61). The literature finds that forced/involuntary commitments to psychiatric institutions increase selectively among adult African American men in times of economic uncertainty, providing evidence of uncovering due to reduced tolerance (22). Prior research also shows that economic contractions may provoke or uncover mental disorders among low SES African American youth resulting in greater utilization of emergency psychiatry services relative to other races/ethnicities (21). However, these studies predate the Great Recession of 2008 that was deeper and longer lasting than all other recessions since World War II (36) and, hence, may have elicited characteristically different responses relative to earlier economic downturns.

An important limitation of this study is the lack of distinction between emergent and non-emergent users of emergency departments. Owing to data constraints in SEDD, we cannot identify whether the observed (relative) increase in publicly insured African American visits arises from true psychiatric emergencies or as a consequence of higher utilization by non-emergent cases. It remains plausible that among low SES minorities, aggregate employment decline may increase non-emergent ED usage (not limited to psychiatric visits only) (62), without increasing the incidence or severity of illnesses. Conversely, prior research also shows that utilization of routine (non-urgent) psychiatric services declines among African American youth (relative to white) after ambient economic shocks, suggesting that while white youth may increase service utilization for routine psychiatric care (including therapy and counseling), African Americans experience a higher proportion of “true emergencies”, tilting the overall rates of PREDVs in their direction (21). Future research may extend this study by analyzing ED-based psychiatric inpatient admissions from the State Inpatient Database (63) to determine whether racial disparities in PREDVs increase among both inpatient (indicating severe cases) and outpatient (relatively less severe) ED visitors or are limited to specific types of PREDVs only.

We also do not have *a priori* hypotheses for the “help-seeking incubation period” (i.e., monthly exposure lags) of different illnesses following aggregate employment decline. Hence, we do not test differential responses to the exposure by illness “type” (e.g., mood disorders versus psychoses). While studies observe a rise in clinical depression (64), alcohol abuse (binge drinking) (65), and suicides (66) among vulnerable sociodemographic groups following the 2008 recession, the underlying theory of illness-specific temporal lags remains under-developed, and may be explored in future research.

Our analysis indicates 15 additional African American PREDVs (among the working age, publicly insured) in a county-month following a 10% decline in employment change. The small magnitude of this finding holds more relevance to the theory of dynamic determinants of racial disparities in PREDVs rather than health policy decisions. This modest increase in racial disparities may arise from a combination of “new” singleton ED visitors and/or higher number of revisits among high ED utilizers (i.e., repeat visitors). Each of these two types of ED visitors hold different implications toward our conceptual understanding of who increases their help-seeking following economic downturns, and future research may examine their differential effects on racial disparities in PREDVs.

## Conclusion

Clinicians and the public regard emergency departments as “the safety-net of safety-nets” (67). However, crisis-oriented care offered in Emergency Departments is limited in its ability to ensure the continuity of care and case management required for proper treatment of psychiatric conditions. High psychiatric ED reliance among African Americans reflects the inadequate reach of mental health systems in providing appropriate and timely care to this group. It is plausible that this mental health services “gap” expands during economic recessions when public health agencies face funding shortages and may have to reduce supply (68). Our study shows that low SES African Americans marginally increase psychiatric-related ED utilization than do white individuals during times of ambient economic decline and these findings add to current knowledge of drivers of racial disparities in ED use for mental health.

## Data availability statement

The original contributions presented in the study are included in the article/**Supplementary Material**, further inquiries can be directed to the corresponding author/s.

## Ethics statement

Ethical approval was not required for the study involving humans in accordance with the local legislation and institutional requirements. Written informed consent to participate in this study was not required from the participants or the participants’ legal guardians/next of kin in accordance with the national legislation and the institutional requirements.

## Author contributions

PS: Writing – review & editing, Writing – original draft, Visualization, Validation, Supervision, Software, Project administration, Methodology, Investigation, Formal analysis, Data curation, Conceptualization.
