# Supplementary material for: Macroeconomic antecedents of racial disparities in psychiatric-related emergency department visits
Source: Front Psychiatry. 2024 Jun 12;15:1287791. doi: 10.3389/fpsyt.2024.1287791 (PMC11200170; doi:10.3389/fpsyt.2024.1287791)
Supplement: Supplementary file 1 [file Table_1.docx]

**Appendix**

**Table A.1** List of ICD 9 codes for psychiatric diagnosis (per Clinical Classification Software categories) used for sample selection.

| **Description** | **ICD 9 codes** |
| --- | --- |
| Adjustment disorders | 3090 3091 30922 30923 30924 30928 30929 3093 3094 30982 30983 30989 3099 |
| Anxiety disorders | 29384 30000 30001 30002 30009 30010 30020 30021 30022 30023 30029 3003 3005 30089 3009 3080 3081 3082 3083 3084 3089 30981 3130 3131 31321 31322 3133 31382 31383 |
| Attention-deficit, conduct, and disruptive behavior disorders | 31200 31201 31202 31203 31210 31211 31212 31213 31220 31221 31222 31223 3124 3128 31281 31282 31289 3129 31381 31400 31401 3141 3142 3148 3149 |
| Delirium, dementia, and amnestic and other cognitive disorders | 2900 29010 29011 29012 29013 29020 29021 2903 29040 29041 29042 29043 2908 2909 2930 2931 2940 2941 29410 29411 29420 29421 2948 2949 3100 3102 3108 31081 31089 3109 3310 3311 33111 33119 3312 33182 797 |
| Developmental disorders | 3070 3079 31500 31501 31502 31509 3151 3152 31531 31532 31534 31535 31539 3154 3155 3158 3159 317 3180 3181 3182 319 V400 V401 |
| Disorders usually diagnosed in infancy, childhood, or adolescence | 29900 29901 29910 29911 29980 29981 29990 29991 30720 30721 30722 30723 3073 3076 3077 30921 31323 31389 3139 |
| Impulse control disorders, NEC | 31230 31231 31232 31233 31234 31235 31239 |
| Mood disorders | 29383 29600 29601 29602 29603 29604 29605 29606 29610 29611 29612 29613 29614 29615 29616 29620 29621 29622 29623 29624 29625 29626 29630 29631 29632 29633 29634 29635 29636 29640 29641 29642 29643 29644 29645 29646 29650 29651 29652 29653 29654 29655 29656 29660 29661 29662 29663 29664 29665 29666 2967 29680 29681 29682 29689 29690 29699 3004 311 |
| Personality disorders | 3010 30110 30111 30112 30113 30120 30121 30122 3013 3014 30150 30151 30159 3016 3017 30181 30182 30183 30184 30189 3019 |
| Schizophrenia and other psychotic disorders | 29381 29382 29500 29501 29502 29503 29504 29505 29510 29511 29512 29513 29514 29515 29520 29521 29522 29523 29524 29525 29530 29531 29532 29533 29534 29535 29540 29541 29542 29543 29544 29545 29550 29551 29552 29553 29554 29555 29560 29561 29562 29563 29564 29565 29570 29571 29572 29573 29574 29575 29580 29581 29582 29583 29584 29585 29590 29591 29592 29593 29594 29595 2970 2971 2972 2973 2978 2979 2980 2981 2982 2983 2984 2988 2989 |
| Alcohol-related disorders | 2910 2911 2912 2913 2914 2915 2918 29181 29182 29189 2919 30300 30301 30302 30303 30390 30391 30392 30393 30500 30501 30502 30503 3575 4255 5353 53530 53531 5710 5711 5712 5713 76071 9800 |
| Substance-related disorders | 2920 29211 29212 2922 29281 29282 29283 29284 29285 29289 2929 30400 30401 30402 30403 30410 30411 30412 30413 30420 30421 30422 30423 30430 30431 30432 30433 30440 30441 30442 30443 30450 30451 30452 30453 30460 30461 30462 30463 30470 30471 30472 30473 30480 30481 30482 30483 30490 30491 30492 30493 30520 30521 30522 30523 30530 30531 30532 30533 30540 30541 30542 30543 30550 30551 30552 30553 30560 30561 30562 30563 30570 30571 30572 30573 30580 30581 30582 30583 30590 30591 30592 30593 64830 64831 64832 64833 64834 65550 65551 65553 76072 76073 76075 7795 96500 96501 96502 96509 V6542 |
| Suicide and intentional self-inflicted injury | E9500 E9501 E9502 E9503 E9504 E9505 E9506 E9507 E9508 E9509 E9510 E9511 E9518 E9520 E9521 E9528 E9529 E9530 E9531 E9538 E9539 E954 E9550 E9551 E9552 E9553 E9554 E9555 E9556 E9557 E9559 E956 E9570 E9571 E9572 E9579 E9580 E9581 E9582 E9583 E9584 E9585 E9586 E9587 E9588 E9589 E959 V6284 |
| Screening and history of mental health and substance abuse codes | 3051 30510 30511 30512 30513 33392 7903 V110 V111 V112 V113 V114 V118 V119 V154 V1541 V1542 V1549 V1582 V6285 V663 V701 V702 V7101 V7102 V7109 V790 V791 V792 V793 V798 V799 |
| Miscellaneous mental health disorders | 29389 2939 30011 30012 30013 30014 30015 30016 30019 3006 3007 30081 30082 3021 3022 3023 3024 30250 30251 30252 30253 3026 30270 30271 30272 30273 30274 30275 30276 30279 30281 30282 30283 30284 30285 30289 3029 3060 3061 3062 3063 3064 30650 30651 30652 30653 30659 3066 3067 3068 3069 3071 30740 30741 30742 30743 30744 30745 30746 30747 30748 30749 30750 30751 30752 30753 30754 30759 30780 30781 30789 3101 316 64840 64841 64842 64843 64844 V402 V403 V4031 V4039 V409 V673 |

Source: Healthcare Cost and Utilization Project (HCUP) - Agency for Healthcare Research and Quality, Rockville, MD. Clinical Classifications Software (CCS) for ICD-9-CM. hcup-us.ahrq.gov. URL <https://www.hcup-us.ahrq.gov/toolssoftware/ccs/ccs.jsp>. Last Updated June, 2017. Accessed July, 2018.

**Table A.2** Overall Least Squares (OLS) linear fixed effects regression results predicting log transformed population rates (per 100,000 population) of PREDVs among 18-64 year old publicly insured group as a function of monthly percent employment change lagged by 0 to 3 months (other covariates including not shown) for: white individuals (Model a), African Americans (Model b), and interaction of binary race (African American = 1, white = 0) with monthly percent employment change lagged by 0 to 3 months (Model c). SE = Standard Error.

| **Covariates** | *Model a: White* | | *Model b: African American* | | *Model c: Interaction between exposure and binary race* | |
| --- | --- | --- | --- | --- | --- | --- |
|  | **Coefficient** | **SE** | **Coefficient** | **SE** | **Coefficient** | **SE** |
| Employment change (lag 0) | 0.002 | 0.002 | -0.002 | 0.004 | 0.001 | 0.002 |
| Employment change (lag 1) | -0.003* | 0.002 | -0.002 | 0.003 | -0.003 | 0.002 |
| Employment change (lag 2) | -3.1e-5 | 1.2e-4 | 0.003 | 0.003 | 0.001 | 0.001 |
| Employment change (lag 3) | -9.1e-5 | 1.4e-4 | -0.006* | 0.003 | -1.8e-5 | 0.002 |
| Race (binary, reference = white) | -- | -- | -- | -- | 1.543**** | 0.068 |
| Employment change (lag 0)*Race | -- | -- | -- | -- | -0.002 | 0.005 |
| Employment change (lag 1)*Race | -- | -- | -- | -- | -0.001 | 0.003 |
| Employment change (lag 2)*Race | -- | -- | -- | -- | 0.002 | 0.004 |
| Employment change (lag 3)*Race | -- | -- | -- | -- | -0.007* | 0.003 |
| *p value < 0.1; **p value < 0.05; ***p value < 0.01; ****p value < 0.001 | | | | | | |

**Table A.3** Linear (OLS) regression results predicting African American race (relative to white) among publicly insured PREDVs aged 18-64 years as a function of monthly percent employment change lagged by 0 to 3 months (other covariates not shown).

| **Variables** | **Coefficient** | **95% Confidence Interval** |
| --- | --- | --- |
| Employment change (lag 0) | -2.1e-4 | [-0.001 0.001] |
| Employment change (lag 1) | 1.6e-4 | [-0.001 0.001] |
| Employment change (lag 2) | -2.7e-4 | [-0.001 3.3e-4] |
| Employment change (lag 3) | -0.001* | [-0.001 -4.7e-5] |
| Sex: Female (reference = male) | 0.010* | [0.001 0.019] |
| Age | 0.001 | [-0.001 0.003] |
| Age squared | -3.3e-5* | [-6.4e-5 -1.03e-6] |
| *pvalue < 0.05; **pvalue < 0.01; ***pvalue < 0.001 | | |

**Table A.4** Logistic regression results predicting African American race (relative to white) among publicly insured PREDVs aged 18-64 years, excluding year 2011, as a function of monthly percent employment change lagged by 0 to 3 months (other covariates not shown) (N = 2,224,053).

| **Variables** | **Odds Ratio** | **95% Confidence Interval** |
| --- | --- | --- |
| Employment change (lag 0) | 0.999 | [0.992 1.007] |
| Employment change (lag 1) | 1.001 | [0.993 1.009] |
| Employment change (lag 2) | 0.995 | [0.990 1.001] |
| Employment change (lag 3) | 0.994* | [0.989 0.999] |
| Sex: Female (reference = male) | 1.060* | [1.007 1.115] |
| Age | 1.006 | [0.994 1.018] |
| Age squared | 1.000* | [1.000 1.000] |
| *pvalue < 0.05; **pvalue < 0.01; ***pvalue < 0.001 | | |

**Table A.5** Logistic regression results predicting African American race (relative to white) among publicly insured PREDVs aged 18-64 years, as a function of monthly percent employment decline shocks lagged by 0 to 3 months (other covariates not shown).

| **Variables** | **Odds ratio** | **95% Confidence Interval** |
| --- | --- | --- |
| Employment shock at lag 0 | 0.996 | [0.985 1.007] |
| Employment shock at lag 1 | 1.009 | [0.995 1.023] |
| Employment shock at lag 2 | 0.997 | [0.988 1.005] |
| Employment shock at lag 3 | 0.987** | [0.978 0.996] |
| Sex: Female (reference = male) | 1.067* | [1.013 1.124] |
| Age | 1.004 | [0.992 1.016] |
| Age squared | 1.000* | [1.000 1.000] |
| *pvalue < 0.05; **pvalue < 0.01; ***pvalue < 0.001 | | |
